# Supplementary material for: Inhibition of Gastrin-Releasing Peptide Attenuates Phosphate-Induced Vascular Calcification
Source: Cells. 2020 Mar 17;9(3):737. doi: 10.3390/cells9030737 (PMC7140688; doi:10.3390/cells9030737)
Supplement: Supplementary file 1 [file cells-09-00737-s001.pdf]

cells

Inhibition of Gastrin-Releasing Peptide Attenuates Phosphate-Induced Vascular Calcification

Hyun-Joo Park [1,2], Yeon Kim [1,2], Mi-Kyoung Kim [1], Jae Joon Hwang [3], Hyung Joon Kim [1,2], Soo-Kyung Bae [2,4] and Moon-Kyoung Bae [1,2,*]

1 Department of Oral Physiology, BK21 PLUS Project, School of Dentistry, Pusan National University, Yangsan 50610, Korea; phj3421@hanmail.net (H.-J.P.); graceyeon88@gmail.com (Y.K.); eenga@naver.com (M.-K.K.); hjoonkim@pusan.ac.kr (H.J.K)
2 Dental and Life Science Institute, School of Dentistry, Pusan National University, Yangsan 50610, Korea; skbae@pusan.ac.kr (S.-K.B.)
3 Department of Oral and Maxillofacial Radiology, School of Dentistry, Pusan National University, Dental Research Institute, Yangsan 50610, Korea; softdent@pusan.ac.kr (J.J.H)
4 Department of Dental Pharmacology, BK21 PLUS Project, School of Dentistry, Pusan National University, Yangsan 50610, Korea
* Correspondence: mkbae@pusan.ac.kr (M.-K.B.); Tel.: +82-51-510-8239

Supplementary Information

The following file contains supplementary material for the paper "Inhibition of gastrin-releasing peptide attenuates phosphate-induced vascular calcification".

This file is composed of:

- Supplementary tables (2 tables)
- Supplementary figures with supplementary figure legends (6 figures)

Supplementary Tables.

Table S1. Primer sequences for real-time RT-PCR

	Genes	Accession No.	Sequences (5′ → 3′)	Length(bp)
**Rat**	β-actin	NM_031144.3	Forward: AGGGAAATCGTGCGTGAC<br>Reverse: CGCTCATTGCCGATAGTG	146bp
	GRP	NM_133570.5	Forward: CTGGGAAGAAGCTGCAAGGA<br>Reverse: TCTGGATCCCAAGTAGGCTG	111bp
	GRP-R	NM_012706.2	Forward: TGATTCAGAGTGCCTACAATCTTC<br>Reverse: CTTCCGGGATTCGATCTG	71bp
	Runx2	NM_001278483.1	Forward: GCCGGGAATGATGAGAACTA<br>Reverse: TGGGGAGGATTTGTGAAGAC	155bp
	calponin	D14437.1	Forward: GAACAAGCTGGCCCAGAAAT<br>Reverse: GGCCATCCATGAAGTTGCTC	104bp
**Mouse**	β-actin	BC138614.1	Forward: TGTTACCAACTGGGACGACA<br>Reverse: GGGGTGTTGAAGGTCTCAAA	166bp
	GRP	NM_175012.4	Forward: ACCTCCTAGAAGCTGCTGGG<br>Reverse: GTCGTTGTCCCTTCAGCTGG	185bp
	GRP-R	NM_008177.3	Forward: TGATTCAGAGTGCCTACAATCTTC<br>Reverse: CTTCCGGGATTCGATCTG	71bp
	Runx2	NM_001145920.2	Forward: CTCTGGCCTTCCTCTCTCAG<br>Reverse: GTAGGTAAAGGTGGCTGGGT	150bp
	calponin	BC138864.1	Forward: CGCATCGGGAACAACTTCAT<br>Reverse: GGTGCCAGTTCTGAGTTGAC	118bp
	Bcl2	BC095964.1	Forward: GCCCTGTGGATGACTGAGTA<br>Reverse: CAGGTATGCACCCAGAGTGA	189bp
	Bad	BC006762.1	Forward: AGGGATGGAGGAGGAGCTTA<br>Reverse: GGAACCCTCAAACTCATCGC	121bp

Table S2.

Study groups	n	Body weight (g)	BUN (mg/dl)	Creatinine (mg/dl)	Phosphate (nmole/μl)	Calcium (mg/dl)
sham+NP	5	25.37±0.13	21.64±0.83	0.30±0.01	0.14±0.01	7.71±0.99
sham+HP	5	25.89±0.30	22.56±0.58	0.35±0.04	0.18±0.01#	7.42±1.49
CKD+NP	7	17.76±0.23	94.97±2.99*	1.71±0.19*	0.20±0.02*	7.39±1.24
CKD+HP	7	18.84±0.34	96.56±1.11*	1.83±0.10*	0.31±0.03*#	8.04±1.35
CKD+NP+vehicle	10	17.82±0.24	93.75±3.48	1.75±0.14	0.20±0.01	7.14±1.00
CKD+NP+RC-3095	10	18.44±0.23	92.63±2.64	1.81±0.06	0.15±0.03	7.31±0.99
CKD+HP+vehicle	10	18.10±0.40	101.64±0.85	1.84±0.15	0.30±0.03**	7.53±1.01
CKD+HP+RC-3095	10	18.14±0.44	99.25±3.35	1.79±0.14	0.24±0.01**##	7.59±1.33

Table S2. Physical and biochemical parameters in CKD mice. CKD mice were fed with a normal phosphate (0.5%, NP) or high phosphate (1.8%, HP) diet for 12 weeks. For RC-3095 treatment, CKD mice were injected with RC-3095 (1 mg/kg) or vehicle (0.9% saline) three times a week for a total 12 weeks. Body weight, and the serum levels of blood urea nitrogen (BUN), creatinine, phosphate, and calcium in each group of mice were measured using colorimetric assay kits. *P<0.01 vs. sham-CKD, **P<0.01 vs. CKD+NP, #P<0.05 vs. NP, ##P<0.01 vs. vehicle. Data shown are the mean ± SD, obtained for at least three independent experiments.

Supplementary Figures

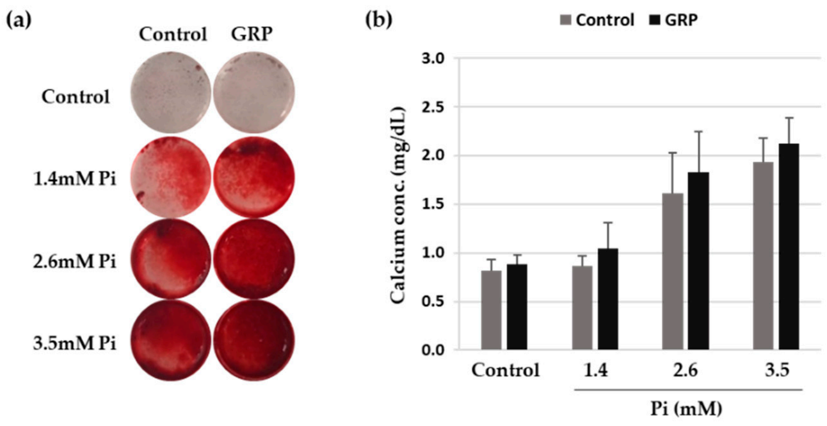

Figure 1. Effect of exogenous GRP_{1-27} on Pi-induced calcification of A7r5 cells. A7r5 cells were treated with GRP for 24 h. A7r5 cells were cultured in calcification medium (1.4, 2.6, and 3.5 mM Pi) with or without GRP (1μM). After 5 days in culture, VSMC calcification was determined by ARS staining (**a**) and calcium content assay (**b**).

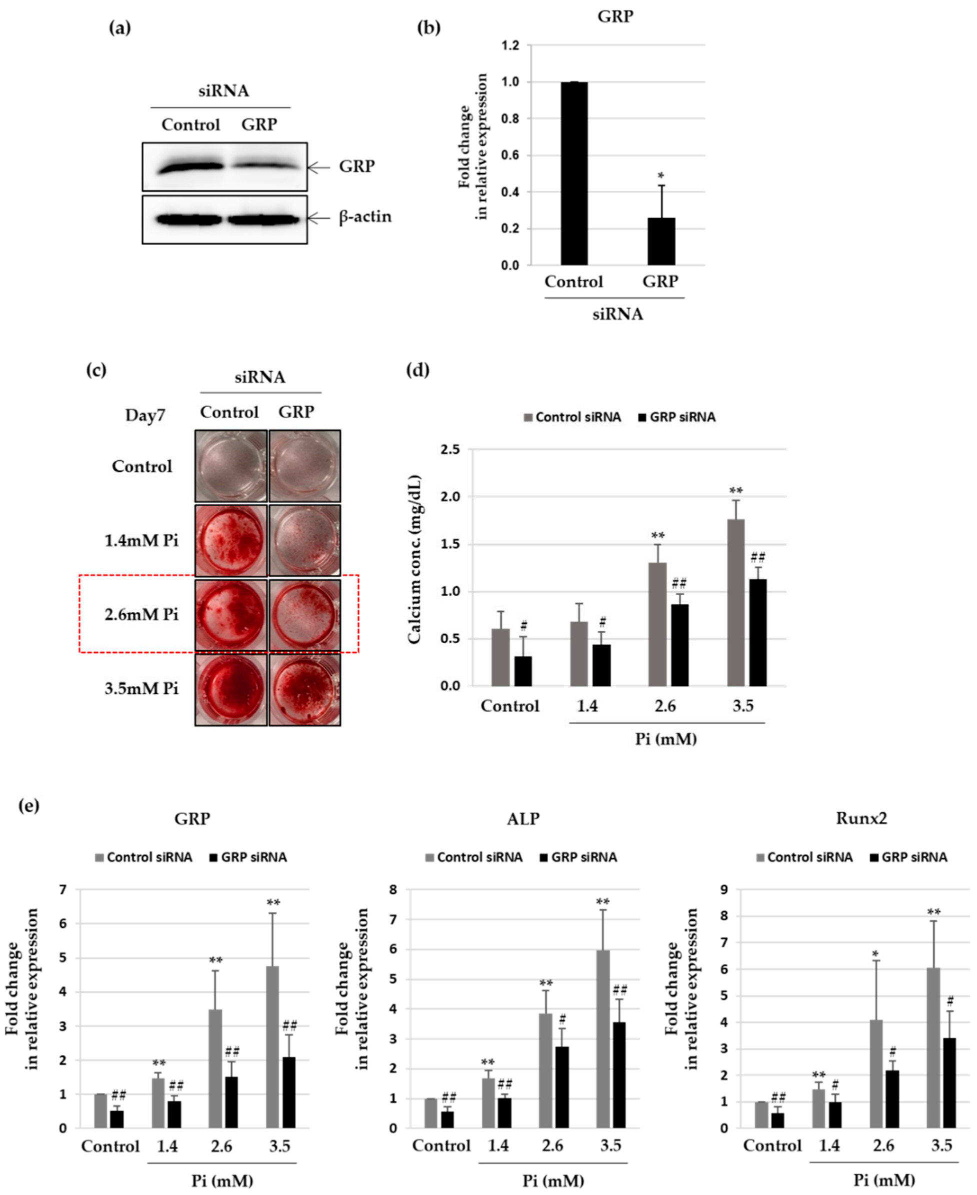

Figure S2. Effect of *GRP* knockdown on Pi-induced calcification of A7r5 cells. A7r5 cells were transfected with GRP siRNA or negative control siRNA for 24 h. (**a** and **b**) GRP protein level was examined by western blotting using anti-GRP antibodies. β-actin served as the loading control. *P<0.01 vs. control siRNA. VSMC calcifications at the indicated Pi concentrations were determined by ARS staining (**c**) and calcium content assay (**d**). (**e**) Using real-time RT-PCR, the expression level of GRP, ALP, and Runx2 mRNA was also quantified. The expression level of these genes in the control (untreated) was set to 1, and the values were normalized to the β-actin mRNA levels. *P<0.05; **P<0.01 vs. control. #P<0.05; ##P<0.01 vs. control siRNA. Data shown are the mean ± SD, obtained for at least three independent experiments.

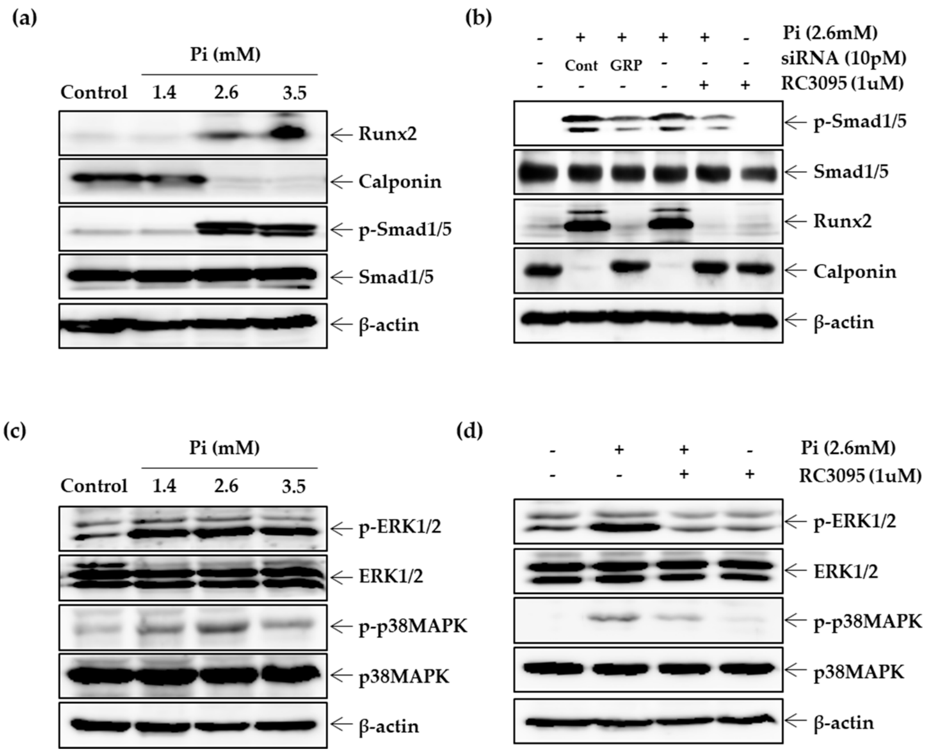

Figure S3. Effect of GRP inhibition on Pi-induced calcification markers and activation of Smad 1/5 and ERK/MAPK pathways in VSMCs. (**a** and **c**) Primary VSMCs were cultured in calcification medium (1.4, 2.6, and 3.5 mM) for 5 days. Western blots were individually probed with antibodies against Runx2, calponin, p-Smad1/5, Smad1/5, p-ERK1/2, ERK1/2, p-p38MAPK, p38MAPK, and β-actin. (**b**) A7r5 cells transfected with GRP siRNA or negative control siRNA for 24 h, and cultured in calcification medium (2.6 mM) for 5 days. (**b** and **d**) Primary VSMCs were cultured in calcification medium with or without RC-3095 (1 μM) for 5 days. Western blots were individually probed with antibodies against Runx2, calponin, p-Smad1/5, Smad1/5, p-ERK, ERK, p-p38, p38, and β-actin.

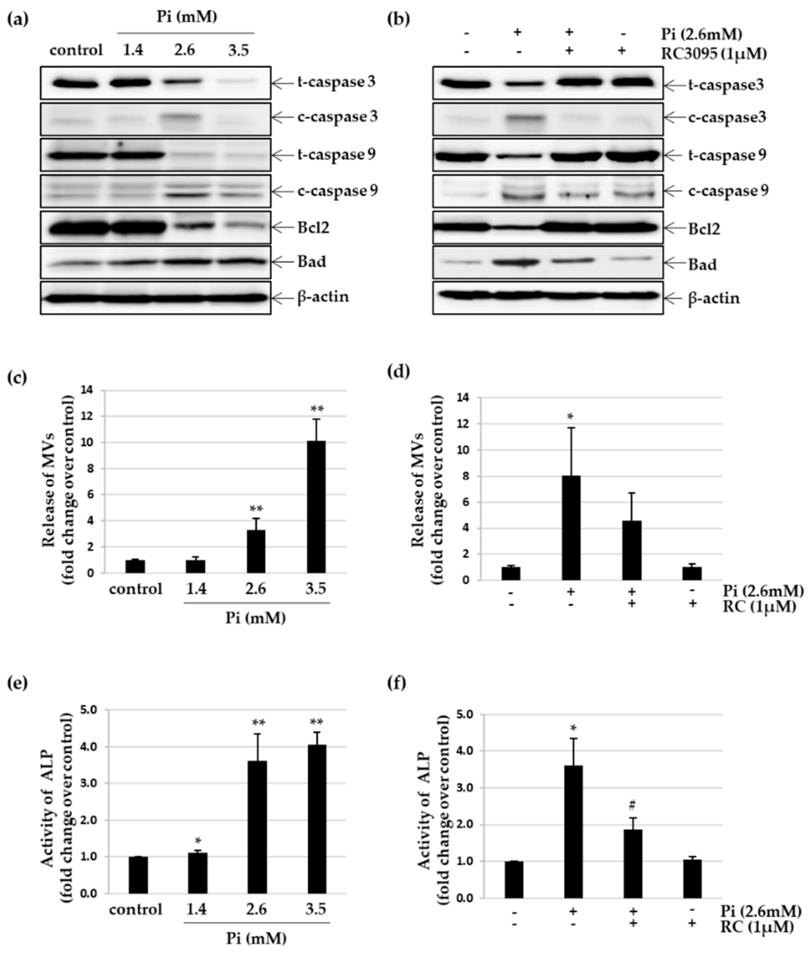

Figure S4. Effect of RC-3095 on Pi-induced apoptosis and matrix vesicle release in VSMCs. (**a**, **c** and **e**) Primary VSMCs were cultured in calcification medium for 7 days. (**a**) Western blots were probed individually with antibodies against total/cleaved caspase-3, total/cleaved caspase-9, Bcl2, Bad, and β-actin. (**b**, **d** and **f**) Primary VSMCs were cultured in calcification medium (2.6 mM) with or without RC-3095 (1 μM) for 7 days. Western blots were probed individually with antibodies against total/cleaved caspase-3, total/cleaved caspase-9, Bcl2, Bad, and β-actin. (**c** and **d**) Matrix vesicles were isolated as described in the Methods. *P<0.05; **P<0.01 vs. control. (**e** and **f**) ALP activity was measured and normalized to total matrix vesicle protein content. *P<0.05; **P<0.01 vs. control, #P<0.01 vs. 2.6mM Pi. Data shown are the mean ± SD, obtained for at least three independent experiments.

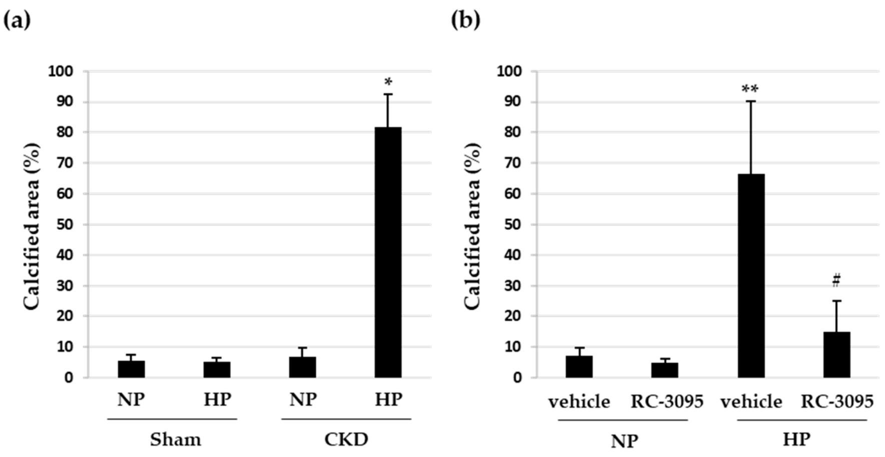

Figure S5. Effect of RC-3095 on vascular calcification in HP-fed CKD mouse. (**a**) CKD mice were fed with a normal phosphate (0.5%, NP) or high phosphate (1.8%, HP) diet. (**b**) For RC-3095 treatment, CKD mice were intraperitoneally injected with RC-3095 (1 mg/kg) or vehicle (0.9% saline) 3 times a week for a total 12 weeks. The percent calcified area in the CKD aorta was calculated using Calcification Analyzer Ver2. *P<0.01 vs. sham-CKD, **P<0.01 vs. CKD+NP, #P<0.01 vs. vehicle. Data shown are the mean ± SD, obtained for at least three independent experiments.

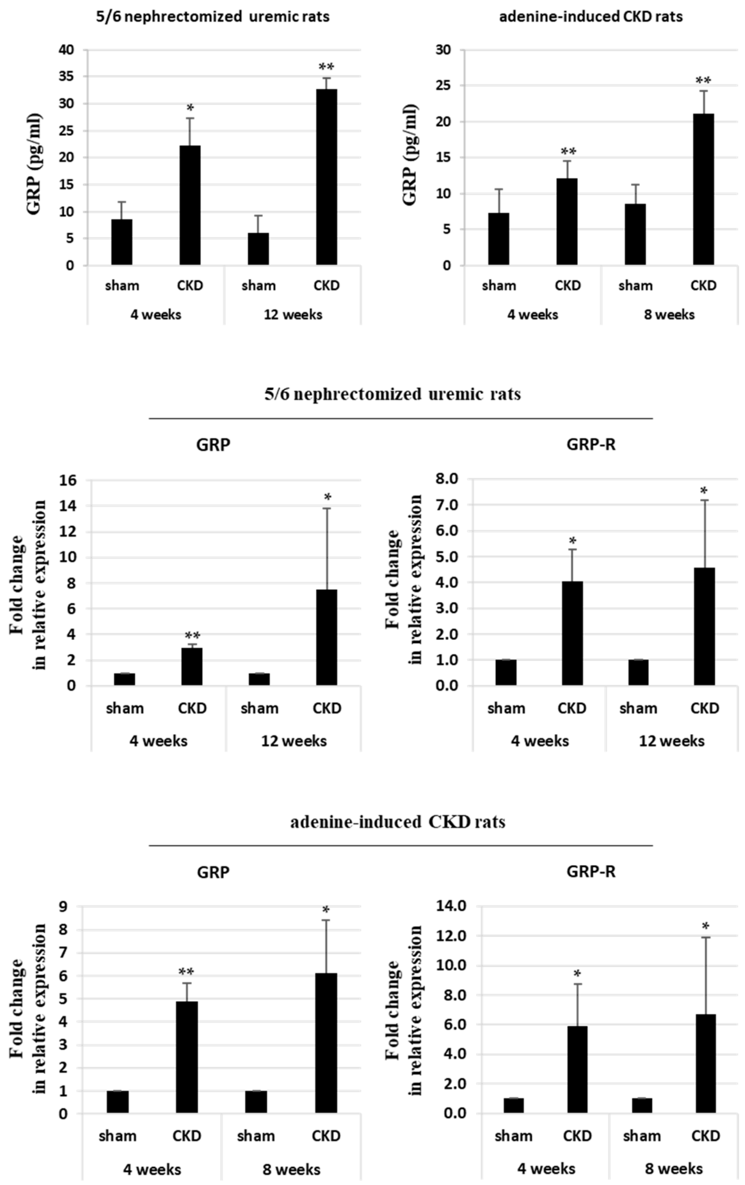

Figure S6. GRP level in plasma and expression of GRP and GRP receptor in aorta from CKD rat model**.** For the 5/6 nephrectomized uremic rat models, renal artery ligation of the right kidney followed by unilateral nephrectomy were performed. For the adenine-induced CKD models, control rats were fed standard chow, while CKD rats were fed standard chow containing 0.75% adenine for 4 or 8 weeks. (**a**) Plasma GRP level was determined by ELISA assay. (**b** and **c**) Total RNA was isolated and analysed by real-time RT-PCR using the specific primers for rat *GRP* and *GRP-R*. The expression level of these genes in the control (untreated) was set to 1, and the values were normalized to the β-actin mRNA levels. *P<0.05; **P<0.01 vs. sham. Data shown are the mean ± SD, obtained for at least three independent experiments.
